# p53-dependent CD51 expression contributes to characteristics of cancer stem cells in prostate cancer

**DOI:** 10.1038/s41419-018-0541-x

**Published:** 2018-05-09

**Authors:** Xin Sui, Jianye Cai, Hongyu Li, Chenchen He, Congya Zhou, Yiping Dong, Li Chen, Bin Zhang, Yingnan Wang, Yanan Zhang, Yuan Qiu, Yuanyuan Zhang, Yang Zhao, Yinong Huang, Yutian Shen, Haoxiang Wu, Jiaqi Xiao, Clifford Mason, Qing Zhu, Suxia Han

**Affiliations:** 1 Department of Oncology, The First Affiliated Hospital, Xi’an Jiaotong University, Xi’an, Shaanxi China; 2 Department of Hepatic Surgery, The Third Affiliated Hospital of Sun Yat-sen University, Guangzhou, Guangdong China; 3 Center for Stem Cell Biology and Tissue Engineering, Key Laboratory for Stem Cells and Tissue Engineering, Ministry of Education, Sun Yat-Sen University, Guangzhou, Guangdong China; 4 Department of Medical Oncology, Collaborative Innovation Center for Cancer Medicine, State Key Laboratory of Oncology in South China, Sun Yat-sen University Cancer Center, Guangzhou, Guangdong China; 5 Department of Oncology, The Second Affiliated Hospital of Xi’an Jiaotong University, Xi’an, Shaanxi China; 6 Guangzhou Cellgenes Biotechnology Co., Ltd., Guangzhou, Guangdong, China; 7 Department of Obstetrics and Gynecology, University of Kansas School of Medicine, Kansas City, KS USA; 8 Department of Abdominal Cancer, West China School of Medicine/ West China Hospital, Sichuan University, Sichuan Chengdu, China

## Abstract

Castration-resistant prostate cancer (CRPC), which is considered to contain cancer stem cells (CSCs), leads to a high relapse rate in patients with prostate cancer (PCa). However, the markers of prostate CSCs are controversial. Here we demonstrate that CD51, in part, correlates with the poor prognosis of PCa patients. Further, we find that CD51 is a functional molecule that is able to promote the malignancy of PCa through enhancing tumor initiation, metastatic potential, and chemoresistance. Moreover, we find that elevated CD51 expression in PCa specimens correlates with p53 loss of function. Mechanistically, we demonstrate that p53 acts via Sp1/3 to repress CD51 transcription, and CD51 is required for PCa stemness and metastasis properties, and is downregulated by p53. Taken together, these results indicate that CD51 is a novel functional marker for PCa, which may provide a therapeutic target for the efficiently restricting PCa progression.

## Introduction

Prostate cancer (PCa) is one of the most common male malignancies in the world^1^. PCa initially produces favorable clinical responses through surgery, radiation therapy, and androgen deprivation. As a heterogeneous disease^2^, castration resistance eventually develops in PCa patients who relapse^3^. The cellular origin and mechanisms proposed for Castration-resistant prostate cancer (CRPC) remain controversial. A recent study reported the presence of cancer stem cells (CSCs) in CRPC^4^. These CSCs could also provide a reservoir for recurrent disease after therapy, which would require either a preexisting resistant phenotype. There is evidence that stem cell markers, such as Nestin, CD44, and ABCG2, are upregulated at the mRNA level in clinical CRPC samples^5^. According to these findings, CSCs might be responsible for the development of CRPC. Thus, research on CSCs would provide a greater understanding of CRPC. Prostate CSCs share many properties, such as self-renewal^6, 7^ and tumorigenic^8^ and metastatic^9^ abilities, with other cancers. Recent efforts to identify and characterize prostate CSCs demonstrated that the primary PCa cell subpopulation possesses a CD44+, CD133+, and androgen receptor (AR)-negative profile, which is similar to normal human prostate stem cells^10, 11^. However, the debate over the markers of prostate CSCs has not been resolved. Recently, our group has identified that CD51 is a marker for colorectal CSCs. Furthermore, CD51 could bind transforming growth factor beta (TGF-β) receptors^12^. A multicenter phase 1 clinical study recruited 26 progressive CRPC patients with bone metastases after chemotherapy had shown evidence of clinical benefit in some patients, after treating with humanized monoclonal antibody targeting av Integrins (CD51)^13^. These findings indicated that CD51 could be a functional surface marker for prostate CSC. As consensus, CSCs share properties and surface markers with normal tissue stem cells^14^. In previous study, our group has demonstrated that the expression of CD51 is synchronized with Nestin in Leydig stem cells^15^. Interestingly, Tschaharganeh et al. showed that p53 restricts expression of the stem and progenitor-cell-associated protein Nestin which is required for tumor initiation in vivo^16^. Recent studies have shown that p53 serves as a barrier to CSC formation by preventing processes, such as dedifferentiation and the formation of damaged stem cells^17^. Considering the role of CD51 in retaining the phenotype of stemness and promoting metastatic process, we hypothesize p53 participate the regulation of CD51 expression in PCa. Consequently, CD51 overexpression, on account of p53 loss, enables the emergence of PCa cells with stem-like properties that are associated with metastasis. Our results reveal an important role for p53 in inhibiting the maintenance of the stem-like state of cancer cells and restricting metastasis.

## Material and methods

### Human patient samples

Human PCa tissue samples were provided by the First Affiliated Hospital of Xi’an Jiaotong University and were diagnosed by a professional pathologist. mRNA array data from human PCa were supplied by The Cancer Genome Atlas (TCGA) (http://cancergenome.nih.gov/). The statistical comparison between the two groups in Table 1 was performed with a two-tailed Student’s *t*-test. Differences in characteristics between the two groups were examined by *χ*2 and Fisher’s exact tests.

**Table 1:** The clinical and pathologic characteristics of 195 PCa patients

Parameter	Overall	CD51+	CD51−	*p*-value
Patients (no)	195	137	58	
Age at diagnosis (year), median (quartile)	70 (63, 75)	70 (63, 76)	70 (64, 73)	0.4264
FU time (months), median (quartile)	21 (17, 39)	20(16, 32.25)	24 (18, 47)	0.2286
PSA level at diagnosis (ng/mL)				
Total, (mean ± SD)	202.7 ± 50.46	257.2 ± 71.00	85.12 ± 40.10	0.1129
≤4	12	6	6	0.1953
4 < PSA ≤ 10	22	14	8	
>10	161	117	44	
Gleason score				
<7	47	14	33	<0.001
3 + 4 = 7	56	37	19	
4 + 3 = 7	46	44	2	
>7	42	40	2	
Unknown	4	2	2	
PT stage				
T0	2	1	1	0.0061
T1	40	21	19	
T2	43	26	17	
T3	30	24	6	
T4	80	65	15	
Lymph node				
N−	129	84	45	0.0323
N+	23	21	2	
Unknown	43	32	11	
Metastasis				
M0	121	78	43	0.0065
M1	30	27	3	

*FU* follow-up, *PSA* prostate-specific antigen, *PT* pathologic tumor

### Cell culture, transfection, and lentiviral transduction

The highly metastatic prostate cell lines DU 145, PC-3, and LNCaP were cultured in complete RPMI medium with 10% fetal bovine serum (FBS; Invitrogen). Lentiviral-mediated short hairpin RNA (shRNA) interference was performed as previously described^18^. CD51 expression was knocked down in PCa cells by transfection with a lentiviral vector expressing an shRNA (Table S1). Lentiviruses were obtained by transfection of 293 cells. PCa cells were seeded in 6-well plates and transfected with shRNA using X-treme GENE HP reagent (Roche). Before experimentation, GFP-positive cells were purified by flow cytometry. The knockdown efficacy of each shRNA-containing lentivirus was assessed after 3 days by western blotting.

### Experimental animals

PCa cells were sorted by CD51, mixed with PBS, and injected subcutaneously into 6–8-week-old SCID mice (Vital River, Beijing, China, http://www.vitalriver.com.cn/). The size of the subcutaneous tumor was recorded on days 7, 14, and 21. After 3 weeks, the mice were killed, and the tumor tissue were weighed, and fixed with formalin. The sections of the xenografts were stained with H&E.

For PCa transplantation studies, cells were injected subcutaneously into 6-week-old male SCID mice in saline after being sorted by CD51 expression. The incidence and size of subcutaneous tumors were monitored for 8 weeks. The sections of the xenografts (5-μm thick) were stained with H&E. Mice that were injected with tumor cells but that did not show any significant neoplasms were sacrificed, and then the injection site was investigated to confirm that the mouse was tumor free. CSC frequency in the samples was determined using the ELDA webtool.

### Flow cytometry

For FACS, cells were analyzed on a FACS flow cytometer (BD, INFLUX, USA) using Spigot 6.1 Software. The purity of the isolated subpopulations regularly exceeded 90%. All FACS analyses and sorting were paired with matched isotype controls, and three independent experiments were performed. The antibodies used include FITC anti-human CD51 (Biolegend, # 327908, 1:200), PE anti-human CD51 (Biolegend, # 327910, 1:200), and PerCP-Cy™ 5.5 mouse anti-Human CD44 (BD Biosciences, #560531, 1:200). Cells (0.5 × 10^6^) were mixed with fluorescently tagged antibodies and incubated in the dark for 15 min at 4 °C. Following incubation, cells were washed with PBS and centrifuged for 5 min at 2000 rpm. After aspiration, approximately 100 μL of cells were suspended in PBS and underwent fluorescence-activated cell sorter analysis.

### Immunohistochemistry (IHC) and immunofluorescence

Immunohistochemical staining of formalin-fixed, paraffin-embedded tissues was performed as previously described^9^, antigen retrieval was performed in EDTA buffer with pH 8.0 at 95 °C for 20 min. Sections were blocked in normal goat serum diluted in PBS and incubated with primary antibody at 4 °C overnight. Biotin-conjugated anti-rabbit or anti-mouse immunoglobulin G was applied, followed by avidin-biotin peroxidase detection system with the DAB substrate. Then, the cells were counterstained with hematoxylin and eosin. The primary antibodies used in IHC were rabbit anti-CD51 (Abcam, Cambridge, MA, #112487, 1:200) and mouse anti-p53 (Santa Cruz Biotechnology, CA, #sc-126, 1:50). IHC-stained tumor sections were examined for positively stained tumor cells and signal intensity, and were scored independently by two observers. The proportion of tumor cells was graded as follows: 0, no positive tumor cells; 1, <10% positive tumor cells; 2, 10–50% positive tumor cells; 3, >50% positive tumor cells. The intensity of staining was scored according to the following criteria: 0, no staining; 1, weak staining; 2, moderate staining; and 3, strong staining. The staining index (SI) score was calculated as the product of the staining intensity score and proportion of positive tumor cells. The IHC staining level was described by SI scores of 0, 1, 2, 3, 4, 6, and 9. Samples with an SI of 0 and 1 were considered negative; all others were positive.

Immunofluorescence was determined as described by Tamada et al.^19^, fixed cells were blocked with normal goat serum in PBS and incubated in primary antibody overnight at 4 °C, followed by incubation with the secondary antibody at room temperature for 1 h. Fixed cells were blocked with 5% normal goat serum (sections) in PBS and incubated in primary antibody (mouse anti-CD51, Santa Cruz Biotechnology, CA, sc-9969, 1:100; rabbit anti-p53, Cell Signaling Technology, MA, #2527, 1:100) overnight at 4 °C, followed by incubation with the secondary antibody (goat anti-mouse IgG Alexa Fluor 555, #A21422; goat anti-rabbit IgG Alexa Fluor 647, #A27040, Thermo Fisher Scientific) at a 1:200 dilution at room temperature for 1 h. Samples were imaged on a Zeiss LSM780 confocal microscope using ZEISS ZEN Imaging Software.

### Immunoblotting

Standard western blotting procedures were performed as described by Henderson et al.^20^. The following antibodies were used: rabbit anti-CD51 (Abcam, Cambridge, MA, #112487, 1:1000); rabbit anti-p53 (Cell Signaling Technology, MA, #2527, 1:1000); rabbit anti-GAPDH (Cell Signaling Technology, MA, #2118 S, 1:1000); mouse anti-SP1 (Santa Cruz Biotechnology, CA, #sc-420, 1:500); mouse anti-SP3 (Santa Cruz Biotechnology, CA, #sc-136479, 1:500); mouse anti-Nestin (BD Biosciences, #611659, 1:1000); rabbit anti-Sox2 (Abcam, Cambridge, MA, #ab97959, 1:500)

### **Reporter constructs and luciferase reporter assays**

The pGL3-basic luciferase reporter plasmid and pRL-TK *Renilla* plasmid were purchased from Promega Corporation (Promega, Madison, WI, USA). The CD51 promoter reporter constructs were made as follows. The CD51 promoter was amplified from human genomic DNA isolated from human mesenchymal stem cells (Table S2) using the following primers: forward (5′-CCGCTCGAGGGAACTCCTGAGCCTAAGCGAT-3′) and reverse (5′-GGAAGATCTGGGATGATATTGACCAAATCCTCGACA-3′). The restriction enzymes XhoI and BgIII were used to insert the construct into the pGL3-basic vector. CD51 promoter activity was detected as described previously^21^.

### **Electrophoresis mobility shift assays (EMSAs)**

The probe for the CD51 promoter was predicted and labeled by a Pierce Biotin 3′ End DNA Labeling Kit (# 89818). EMSA was performed using a LightShift Chemiluminescent EMSA Kit. A double-stranded probe was labeled by a Pierce Biotin 3′ End DNA Labeling Kit. The sense sequences were 5′-CTCGCTGGGGCGGGGGGAGG-3′ (forward) and 5-CCTCCCCCCGCCCCAGCGAG-3 (reverse). The sequences of the mutant proceeds were as follows: 5′-CTCGCTGGGAAGGGGGGAGG-3′ (forward) and 5′-CCTCCCCCCTTCCCAGCGAG-3′ (reverse). The standard Sp1 binding sequences were 5′-ATTCGATCGGGGCGGGGCGAGC-3′ (forward) and 5′-GCTCGCCCCGCCCCGATCGAAT-3′ (reverse). The nuclear protein was affinity purified using a Nucleoprotein Extraction Kit (Sangon, China). EMSA was performed as described previously^22^.

### Sphere-forming assay

DU 145 and PC-3 cells were plated in 96-well ultra-low-attachment plates (Corning) at a density of 1000 viable cells per well. Cells were grown in standard sphere-forming medium (serum-free DMEM/F-12 supplemented with 1 × B27 serum substitute, 20 ng/ml human recombinant epidermal growth factor, and 20 ng/ml basic fibroblast growth factor; all from Invitrogen). Plates were incubated at 37 °C with 5% CO_2_ and cultured for 14 days. Wells with spheres larger than 50 μm in diameter were counted.

### **Colony-forming unit assays**

Cells were suspended in complete RPMI medium and cultured on 100 mm dishes. Additional medium was added, and the cells were cultured for 21 days prior to fixing and staining the colonies with 0.1% crystal violet/PBS. Colonies with diameters of at least 2 mm were counted.

### Side population assays

For the SP assay, in brief, cells were resuspended at 1 × 106 cells/mL in RPMI with 2% FCS, and Hoechst 33342 (Sigma-Aldrich) was added (to a final concentration of 2 mg/mL) and incubated at 37 °C for 90 min. In the control reactions, 100 mM verapamil (Sigma-Aldrich) was added. After the incubation, cells were placed immediately on ice and washed. Hoechst dye dual wavelengths were detected using 450/40 (Hoechst 33342-Blue) and 695/40 (Hoechst 33342-Red) filters. Dead cells were excluded by gating on the forward and side scatter. The data were analyzed by FlowJo software (Ashland, OR, USA).

### Aldehyde dehydrogenase (ALDH)

High ALDH activity is a marker commonly used to isolate stem cells^23^. In brief, we used an Aldefluor Kit (Stem Cell Technologies, #01700). Cells were suspended in Aldefluor assay buffer containing uncharged ALDH1-substrate and BODIPY-aminoacetaldehyde (BAAA) and incubated for 40 min at 37 °C. Living cells take up BAAA and convert it into a negatively charged reaction product, BODIPY-aminoacetate, by intracellular ALDH. BODIPY-aminoacetate was retained inside cells expressing high levels of ALDH and caused the cells to brightly fluoresce. The brightly fluorescent ALDH1-expressing cells (ALDH1-positive cells) were detected in the FITC channel of a FACScan instrument (BD Biosciences). A set of cells with the specific ALDH inhibitor, diethylaminobenzaldehyde (Sigma), served as the negative control for each experiment. Because only cells with an intact cellular membrane can retain the Aldefluor reaction product, only viable ALDH1-positive cells were identified. Cells incubated with BAAA and diethylaminobenzaldehyde were used to establish the baseline fluorescence of these cells and to define the (ALDH1)-positive region. Incubation of cells with the substrate in the absence of diethylaminobenzaldehyde induced a shift in BAAA fluorescence, defining the Aldefluor-positive population. Data were analyzed by using FlowJo. Each experiment was repeated three times.

### Migration assays

Transwell migration assays were performed as described by Bijlsma et al.^24^. A cell suspension of PCa cells in serum-free medium was plated into the upper chamber. The lower chamber was filled with 500 μL of conditioned medium. Cells were cultured at 37 °C with 5% of CO_2_ for 24 or 48 h. Images were acquired under a microscope. Cell counts are expressed as the mean number of cells per field of view. The number of migrated cells was quantified from at least three randomly selected fields from three independent experiments.

The wound-healing assay was performed in the presence of mitomycin C (5 μg/ml). After excluding the influence of cell proliferation, cell monolayers were scratched with a pipette tip and washed twice, and photographs were taken to analyze the percentage of the open wound area at 24 and 48 h (ImageJ software; US National Institutes of Health, Bethesda, MD, USA).

### Time-lapse cell motility assay

The migration of cancer cells was monitored using a Zeiss Axio Observer Z1 microscope over 6 h. Cells were cultured at 37 °C in an enclosed chamber containing a controlled proportion of CO_2_. Cell migration was quantified once every 20 min for 6 h using computer-assisted tracking. The migration distances of individual cells were determined and analyzed.

### **Real-time qPCR**

RT-qPCR experiments were performed as described previously^18^. Total RNA was isolated using Trizol reagent (Invitrogen) according to the manufacturer’s instructions. Two micrograms of total RNA was reverse-transcribed using RevertAid First Strand cDNA Synthesis Kit (Thermo, K1622) according to the manufacturer’s instructions with random hexamers as primers. Quantitative real-time PCR was carried out in a final volume of 20 μl containing cDNA, FastStart Essential DNA Green Master Mix (Roche, 06924204001), and primers specific for GAPDH as an internal control. After incubation at 95 °C for 10 min, products were amplified under the following conditions: 35 cycles of 15 s at 95 °C, 60 s at 60 °C, and 120 s at 50 °C. The designed PCR primers were listed in Table S2.

### **Study approval**

In total, 195 PCa patients were histologically confirmed and were diagnosed according to the American Joint Committee on Cancer. This study was approved by the Ethics Committee of the First Affiliated Hospital of Xi’an Jiaotong University. Written informed consent was obtained from all subjects.

### Statistical analyses

Data presentation and statistical tests are indicated in the figure legends. The statistical comparison between two groups was performed with a two-tailed Student’s *t*-test. All statistical tests were performed on PRISM 6 software. *p* < 0.05 was considered statistically significant.

## Results

### CD51 levels are high in PCa and correlated with a poor prognosis

**Fig. 1:**
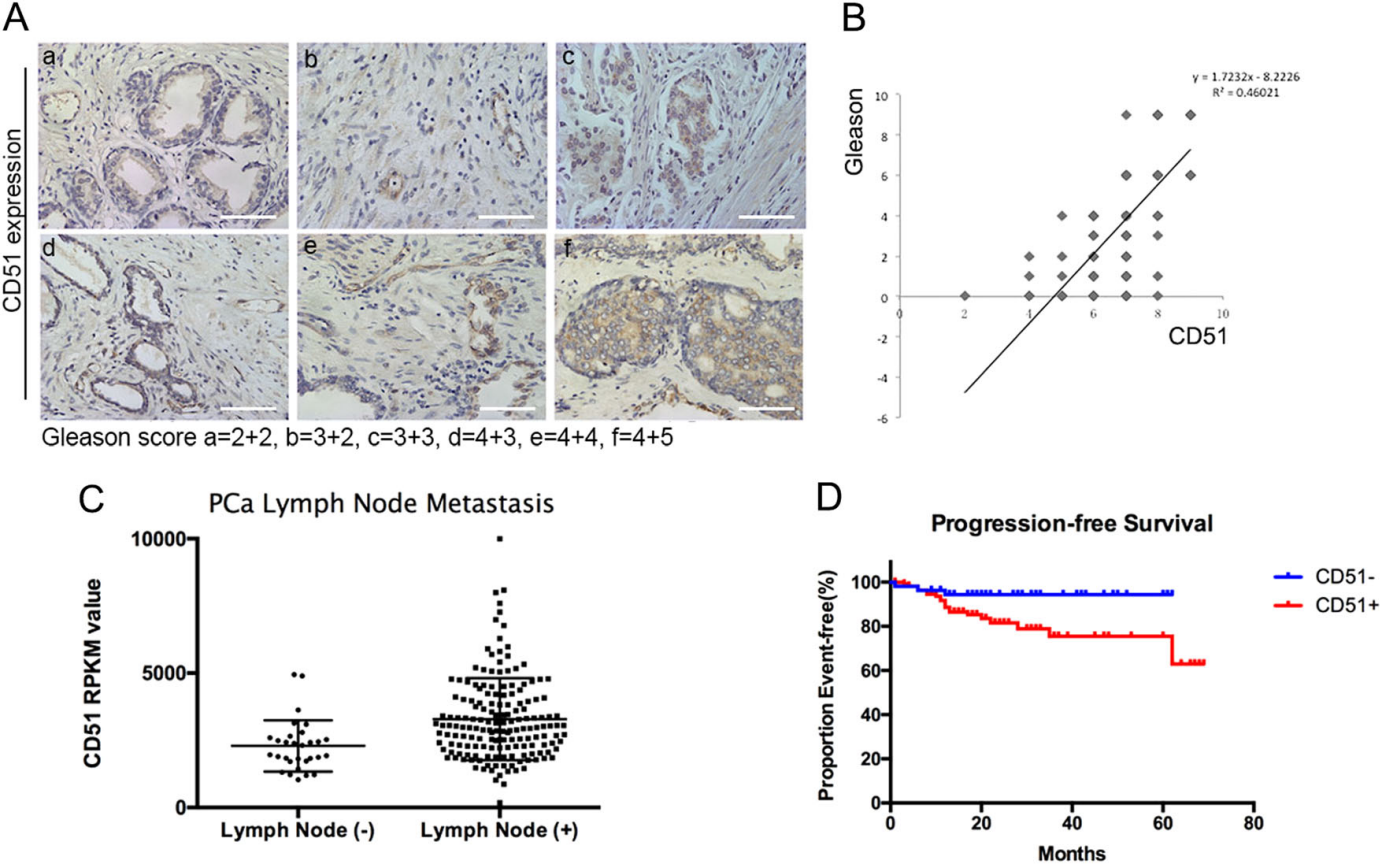
CD51 is associated with the poor outcome of PCa patients. **a** Immunohistochemistry (IHC) analyses showed that CD51 expression level was gradually increased with the Gleason score. Scale bar: 100 μm. **b** Dot plot image showing the positive association of CD51 expression with the Gleason score in PCa, as determined by Spearman correlation. **c** The Cancer Genome Atlas (TCGA) PCa gene expression data set was sorted by CD51 expression and presented in the dot plot image. The top and bottom quartiles (CD51 low and high, respectively) were selected. Positive and negative lymph node metastasis groups were found to have significantly different CD51 expression (*p* *<* 0.001, *t*-test, two tailed). **d** Kaplan–Meier survival analysis of PFS between the CD51+ (*n* = 112) and CD51− (*n* = 54) groups. Median PFS of 20 (CD51+) vs. 24 (CD51−) months for PCa patients (hazard ratio (HR), 0.379; 95% CI, 0.1592–0.9036; *p* *=* 0.028)

A total of 195 PCa patients were enrolled in the study between January 2010 and December 2015. The ages ranged from 44 to 90 (*n* = 195; median (quartile) = 70 (63,75)), and their mean SD serum prostate-specific antigen (PSA) levels at the time of enrollment were 202.7 ± 50.46 ng/mL. The clinical and pathologic characteristics of all 195 PCa patients are shown in Table 1. To investigate if CD51 could predict a poor prognosis, IHC was performed on PCa patient samples. The observation that relatively high CD51 expression was associated with a high Gleason score suggested a positive correlation between CD51 expression and malignant behavior (*r* = 0.68) (Fig. 1a, b). To determine whether CD51 expression correlated with malignant characteristics, such as the tendency for metastasis in PCa samples from other cancer centers, TCGA RNA-seq data were used to match PCa samples to the CD51+ and CD51− subtypes. There was a significant correlation between high CD51 expression and lymph node metastasis (*p* *<* 0.001) (Fig. 1c). Importantly, PCa patients with high CD51 levels displayed a much worse clinical outcome, with a median progression-free survival (PFS) of 20 months compared to 24 months for CD51-negative patients (hazard ratio (HR), 0.379; 95% CI, 0.1592–0.9036; *p* *=* 0.028) (Fig. 1d). Our data suggested that CD51 plays an important role in the progression of PCa.

### **CD51+ PCa is associated with self-renewal capacity**

**Fig. 2:**
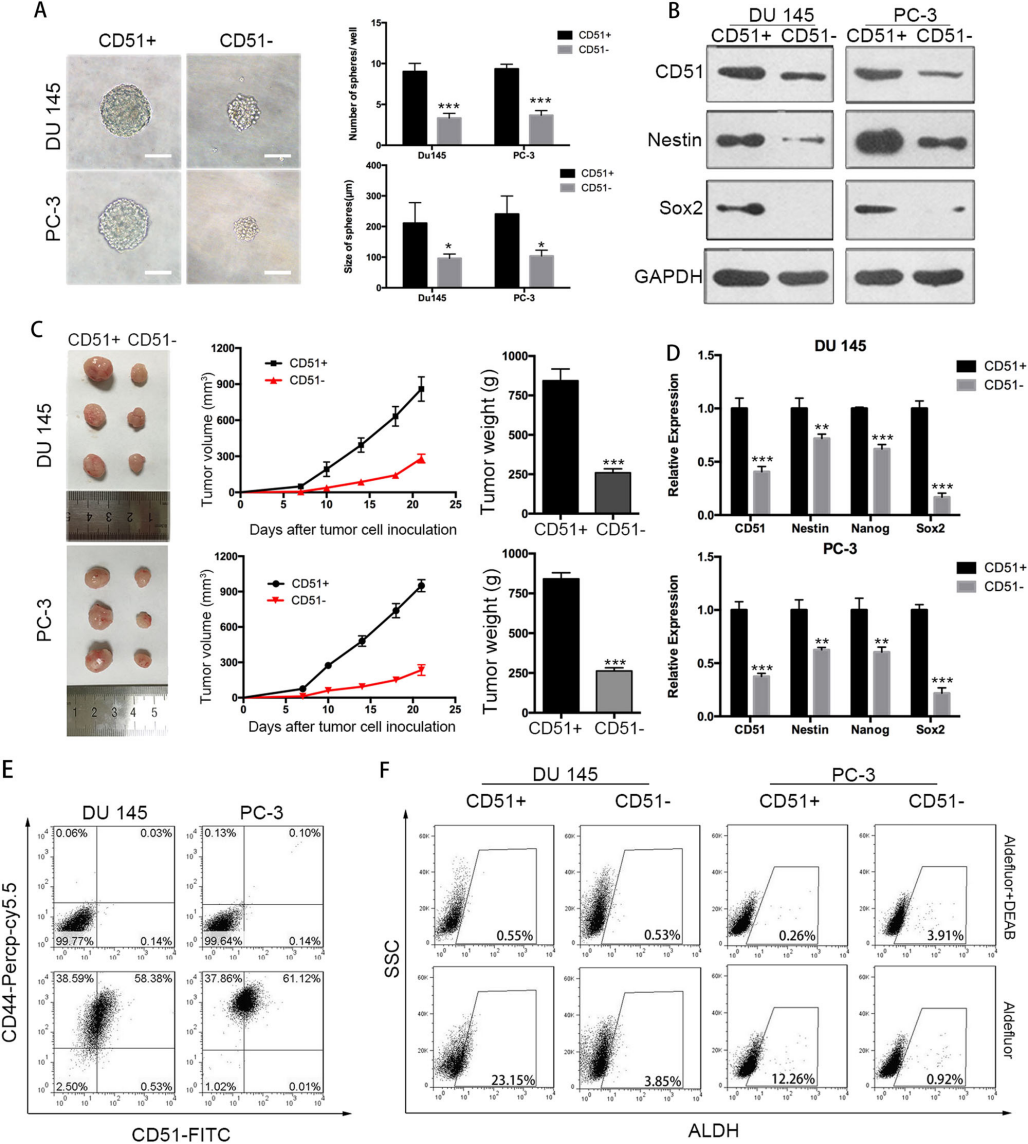
CD51+ cells showed PCa CSC properties. CD51+ or CD51− cells sorted from DU 145 and PC-3 cell lines. **a** Sphere-formation assays showed that the CD51+ cells exhibited higher sphere-forming capacities than the corresponding CD51− cells. Scale bar: 100 μm. **b** CD51, Nestin, and Sox2 immunoblots from CD51+ and CD51− cells in DU 145 and PC-3. **c** Tumor formation in vivo was induced by the subcutaneous injection of PCa cells (dose of 2 × 10^6^) into 6-week-old SCID mice (*n* = 6). Tumors on the left line were formed by CD51+ cells, while tumors on the right line were formed by CD51− cells. **d** RT-qPCR was used to evaluate the expression of the stem-related genes Nestin, Nanog, and Sox2 at the mRNA level in CD51+ and CD51− cells. **e** Flow cytometric analysis of the co-expression of CD51-FITC with the CD44-PerCP-Cy5.5 antibody in two cell lines. **f** Flow cytometric analysis of ALDH activation in CD51+ and CD51− cells. Error bars represent the standard deviation (SD) of data obtained from three independent experiments (**p < 0.01, ***p < 0.001)

Since CD51 was positively associated with the poor prognosis of PCa patients, we wondered if the membrane protein CD51 could serve as a PCa CSC marker. We evaluated the self-renewal ability of CD51+ cells; this ability is the foundational characteristic of CSCs^25^. The AR-negative cell lines PC-3 and DU 145 were used to assess the contribution of CD51 to PCa stem-like behavior. Flow cytometry was used to sort the CD51+ populations in the two PCa cell lines. The ability of cells to form spherical colonies in ultra-low-adherence culture conditions was used to identify CSCs in cancer^26^. Compared to CD51− cells, the CD51+ population formed larger (diameters 174 vs. 115 µm in DU 145, 181 vs. 101 µm in PC-3) and more tumor spheres (Fig. 2a). To test the ability of the CD51+ subset to initiate tumors in vivo, we subcutaneously injected CD51+ or CD51− cells, at a dose of 2 × 10^6^, into the flanks of SCID mice (each group, *n* *=* 6). After 3 weeks, the CD51+ PCa cells yielded tumors with a larger size (859.5 ± 41.36 mm^3^) than the same dose of CD51− cells (281.5 ± 14.70 mm^3^) (Fig. 2c). Furthermore, we tested the expression of stem-related genes, such as Sox2^27^, Nestin^28^, and Nanog^29^, in two groups of cells in two cell lines. The results showed that stem-related genes were higher in the CD51+ population (Fig. 2b, d). To examine whether CD51+ PCa subpopulations display overlapping expression with other proven prostate CSC markers, we performed dual-color flow cytometric analysis for CD44 and CD51 in PCa cell lines. Our results showed that almost all of the PC-3 and DU 145 PCa cells are CD44+ (99.1% in DU145 and 99.9% in PC-3), while only a proportion of cells are CD44+CD51+ (60.2% in DU145 and 61.74% in PC-3) (Fig. 2e). Further, we assessed the presence of a cell population with ALDH enzymatic activity in the CD51+ and CD51− cells; ALDH-positive cells ranged from 23.15% (DU 145) to 12.26% (PC-3) in CD51+ cells, whereas ALDH-positive cells in CD51− cells were low in both cell lines (Fig. 2f). The colony-forming unit assay was used to detect the difference in cell proliferation potential between CD51+ and CD51− cells. Our results showed that the CD51+ subpopulation has a greater ability to form larger colonies in vitro (Fig. S1A). In addition, drug efflux capacity, measured by Hoechst 33342 dye exclusion, was performed with the CD51+ and CD51− subpopulations. Compared with the CD51− group, the percentage of SP was higher in CD51+ cells in the PC-3 group. In addition, SP could not be found in DU 145 cells (Fig. S1B). We also developed orthotopic tumor models in SCID mice. The results showed that CD51+ cells showed higher CSC frequency (Table 2). These data indicate a role for CD51 in PCa initiation.

**Table 2:** In vivo tumor initiation experiments with CD51+ and CD51− subpopulations isolated from the PCa cell lines DU 145 and PC-3

Cell type	Marker	1 × 10^2^	1 × 10^3^	1 × 10^4^	1 × 10^5^	CSC frequency
DU 145	CD51+	0/4	2/4	3/4	4/4	1/4677
	CD51−	0/4	0/4	1/4	3/4	1/62755
	Control	0/4	1/4	2/4	3/4	1/37191
PC-3	CD51+	0/4	2/4	4/4	4/4	1/1609
	CD51−	0/4	0/4	2/4	3/4	1/46872
	Control	0/4	1/4	2/4	4/4	1/10777

### CD51+ cells have increased metastatic and drug-resistant properties

**Fig. 3:**
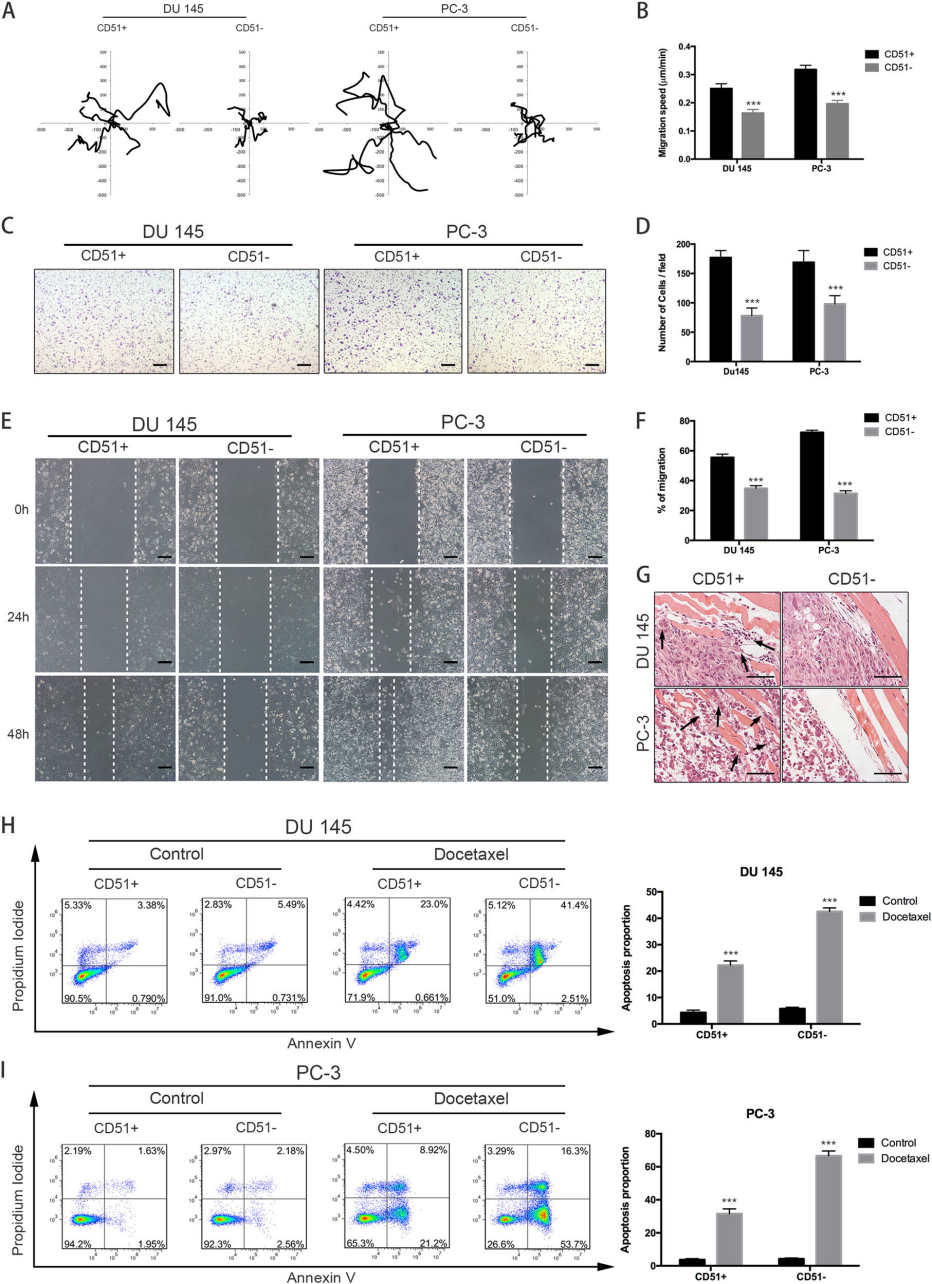
Enhanced migration, invasion, and drug resistance in CD51+ cells. **a** The wind-rose plots for the CD51+ and CD51− cells show differences in metastasis. Cells were tracked over time using semi-automated software. The average velocity of dermal CD51+ cells was 0.250 ± 0.017 µm/min, whereas it was 0.163 ± 0.013 µm/min CD51− cells in the DU 145 cell line. Similarly, it was 0.318 ± 0.015 µm/min vs. 0.197 ± 0.011 µm/min in the PC-3 cell line, respectively. **b** Migration speed was measured and analyzed using a two-tailed *t*-test. Error bars represent the SD from three independent experiments (***p < 0.001). **c** Transwell migration assays showed that CD51+ and CD51− cells migrated toward the chemoattractant. Cell migration was determined after 24 h. Scale bar: 200 µm. **d** Statistical analysis of cell migration. The average was calculated based on three independent experiments. Error bars show the mean ± SD from three technical replicates (two-tailed Student’s *t*-test, ***p *<* 0.001). **e** Wound-healing assay. Phase-contrast images were obtained at 0, 24, and 48 h after scratching the dish. Scale bar: 200 µm. **f** Statistical analysis of cell migration distance. The average was calculated based on three independent experiments. Error bars show the mean ± SD from three technical replicates (two-tailed Student’s *t*-test, ***p *<* 0.001). **g** H&E staining of tumors that formed in SCID mice injected subcutaneously with CD51+ cells confirmed a malignant phenotype in vivo. Scale bar: 100 µm. **h**, **i** Flow cytometric detection of apoptosis and necrosis was performed using annexin V-FITC and propidium iodide (PI). DU 145 (H) and PC-3 (I) cells were divided into three populations: normal viable cells (annexin V and PI negative), early apoptotic cells (annexin V positive and PI negative), and late apoptotic/necrotic cells (annexin V positive and PI positive). Statistical analysis of drug-resistance was performed with a two-tailed *t*-test. The average was calculated based on three independent experiments. Error bars show the mean ± SD from three technical replicates (two-tailed Student’s *t*-test, *p *<* 0.05, **p < 0.01, ***p *<* 0.001)

Previous evidence suggested that prostate CSCs may also promote metastasis and drug-resistance^30^. To determine the cell-intrinsic characteristics of CD51 on motility, a conventional time-lapse cell motility assay was initially performed to track the migration of CD51+ or CD51− cells. Cells were tracked over time using semi-automated software. The average velocity for dermal CD51+ cells was 0.250 ± 0.017 µm/min, whereas the velocity was 0.163 ± 0.013 µm/min in CD51− cells from the DU 145 cell line. Similarly, the velocity was 0.318 ± 0.015 µm/min (CD51+) vs. 0.197 ± 0.011 µm/min (CD51−) in PC-3 cells. Wind-rose plots showed that CD51+ cells migrated further than the CD51− cells during the 6 h of tracking (Fig. 3a, b). Transwell migration assays were performed to examine differences between the migratory behaviors of CD51+ and CD51− PCa cells. The number of cells that migrated through the membrane pores toward the FBS (chemoattractant) on the lower side was counted after 24 h. We observed that CD51+ cells showed higher migration properties compared to CD51− cells (Fig. 3c, d). The wound-healing assay was performed in the presence of mitomycin C and revealed that CD51+ cells possessed greater motility than CD51− cells (Fig. 3e, f). To investigate their invasive ability in vivo, we examined the xenograft tumor specimens described above by H&E staining. The invasive fronts that infiltrated muscle layers were only present in CD51+ xenograft tumors (Fig. 3g).

As one of the key characteristics of CSCs, drug-resistant assays were performed to determine the difference between CD51+ and CD51− cells. After testing the basic IC50 of the two cell lines (Fig. S2A), we used flow cytometry to detect the apoptosis of PCa cells following treatment with docetaxel for 48 h. The results showed that the apoptosis rate induced by docetaxel was significantly higher in CD51− cells compared with the CD51+ cells (Fig. 3h, i). Our data indicate that CD51+ cells have a strong association with migration and drug resistance.

### CD51 is functionally required for PCa stem-related properties

**Fig. 4:**
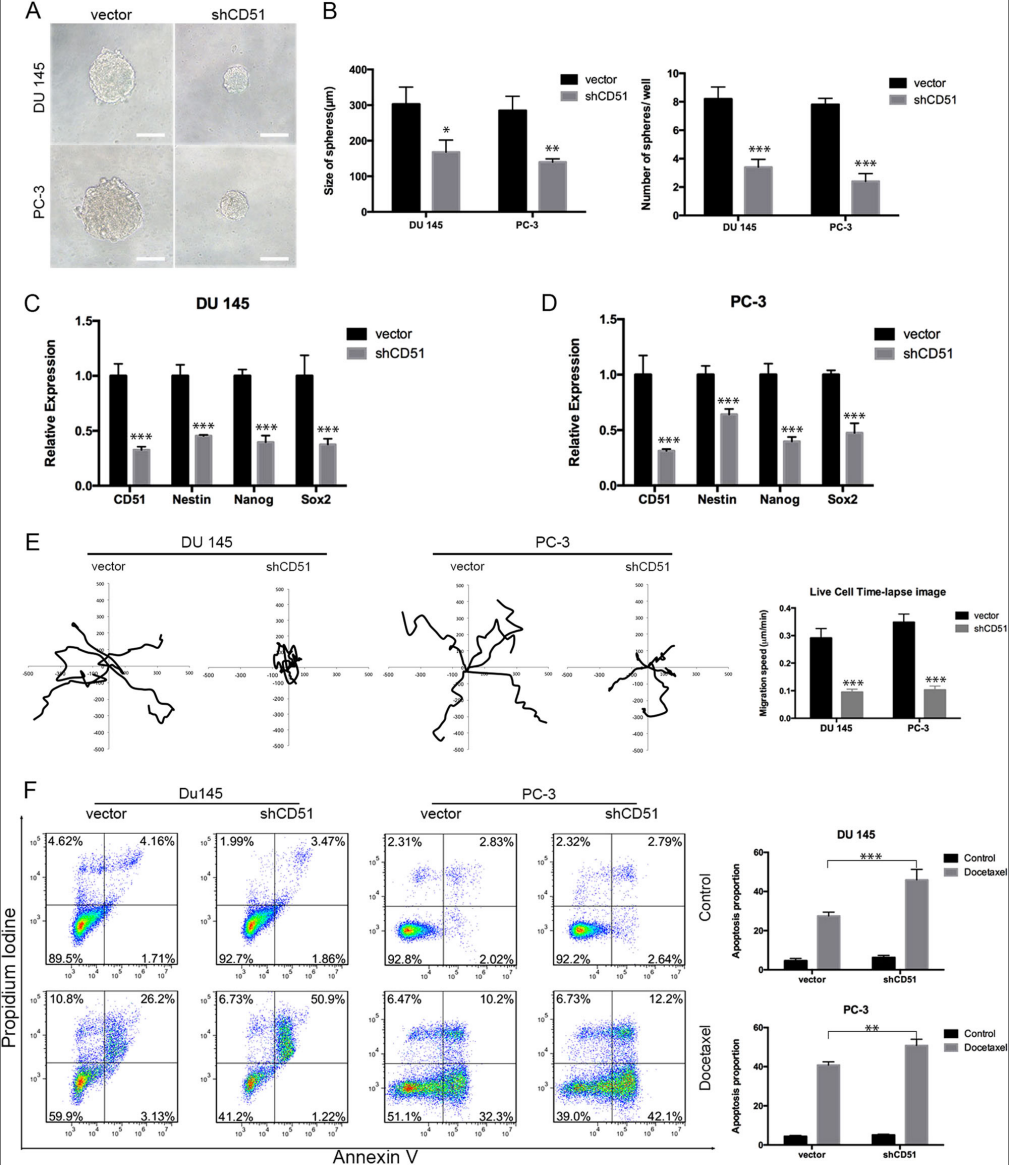
Cells with CD51 knockdown lose their tumor-initiation and metastasis abilities. **a** Sphere-formation assays showed that shCD51 decreased the sphere-forming capacities of both cell lines. Scale bar: 50 μm. **b** Statistical analysis of sphere-formation assays from three independent experiments using *t*-test analysis. **c**, **d** RT-qPCR showed the differences in the mRNA expression level of the stem-related genes Nestin, Nanog, and Sox2 after knocking down CD51 in DU 145 (**c**) and PC-3 (**d**) cells. **e** Wind-rose plots showed that migration ability was repressed after knocking down CD51. Statistical analysis of cell migration speed was determined using a two-tailed *t*-test. **f** Drug-resistance assays were performed using flow cytometry to detect the apoptotic portion (based on Annexin V-FITC binding) of cells after treatment with docetaxel for 48 h. Statistical analysis of drug-resistance was performed with a two-tailed *t*-test. The average was calculated based on three independent experiments. Error bars show the mean ± SD from three technical replicates (two-tailed Student’s *t*-test, *p *<* 0.05, **p < 0.01, ***p *<* 0.001)

To test the function of CD51 in tumor initiation, CD51 expression was reduced using a lentivirus carrying a shRNA against CD51 in DU 145 and PC-3 cells. To confirm if CD51 is essential for maintenance of stem-like properties in 22Rv1 cells (CRPC cell line), analysis of the self-renewal abilities of CD51-knockdown PCa cells were also performed. The efficiency of knocking down CD51 was confirmed by immunoblotting and RT-qPCR (Fig. S2B and S2C). We tested the sphere-forming abilities in vitro; the results showed that knocking down CD51 repressed the self-renewal ability of PCa cells (Fig. 4a, b and S4A). Tumor formation in vivo was also decreased after knocking down CD51 in DU 145, PC-3, and 22Rv1 cells (Fig. S3A, S4B and S4C). We also tested the expression of stem-related genes (Sox2, Nestin, and Nanog) in the DU 145, PC-3, and 22Rv1 cell lines. The results showed that stem-related genes were decreased after the knockdown of CD51 expression (Fig. 4c, d and S4D). To determine the function of CD51 in promoting cell migration, we analyzed the movement distance and speed of the cells by a time-lapse cell motility assay in DU 145 and PC-3 cells after knocking down CD51. The results showed a decrease in the migratory capacities of cells with the shCD51 vector. Wind-rose plots of the representative cell tracks further illustrate that CD51 promotes PCa cell movement in vitro (Fig. 4e). Transwell migration assays in 22Rv1 cells showed that shCD51 cells consistently showed decreased miguration abilities (Fig. S4E). To test whether CD51 is responsible for PCa metastasis in vivo, we injected PC-3 cells labeled with red fluorescent protein into the tail veins of SCID mice (*n* = 6). Mice were killed 6 weeks after the cell injection. Immunofluorescence results showed that PC-3 cells with knocked down CD51 expression were negative for lung metastasis. Multiple small clumps of cells were observed in the lungs when injected with PC-3 cells compared with the control vector (Fig. S3B). Neither control nor DU 145 cells with knocked down CD51 had metastatic lesions in the lung. H&E staining showed less and little tumor mass in lungs with shCD51 PC-3 cells (Fig. S3C). PC-3 cells with shCD51 showed less metastatic potential. The DU 145 cells, with relatively low metastatic capabilities^31^, did not form metastatic foci. H&E staining of sections from PCa tumor formed by 22Rv1 cells showed that knockdown of CD51 depressed PCa invasion property (Fig. S4F). Chemo-resistant ability was detected via testing cell apoptosis rate used flow cytometry after treating with docetaxel, as shown in Fig. 4f and S4G. The results showed that the apoptosis induced by docetaxel was significantly increased in the shCD51 group compared with the vector control group. Our data suggests that CD51 could be required for the malignant expansion of PCa.

### p53 downregulates CD51 expression in PCa

**Fig. 5:**
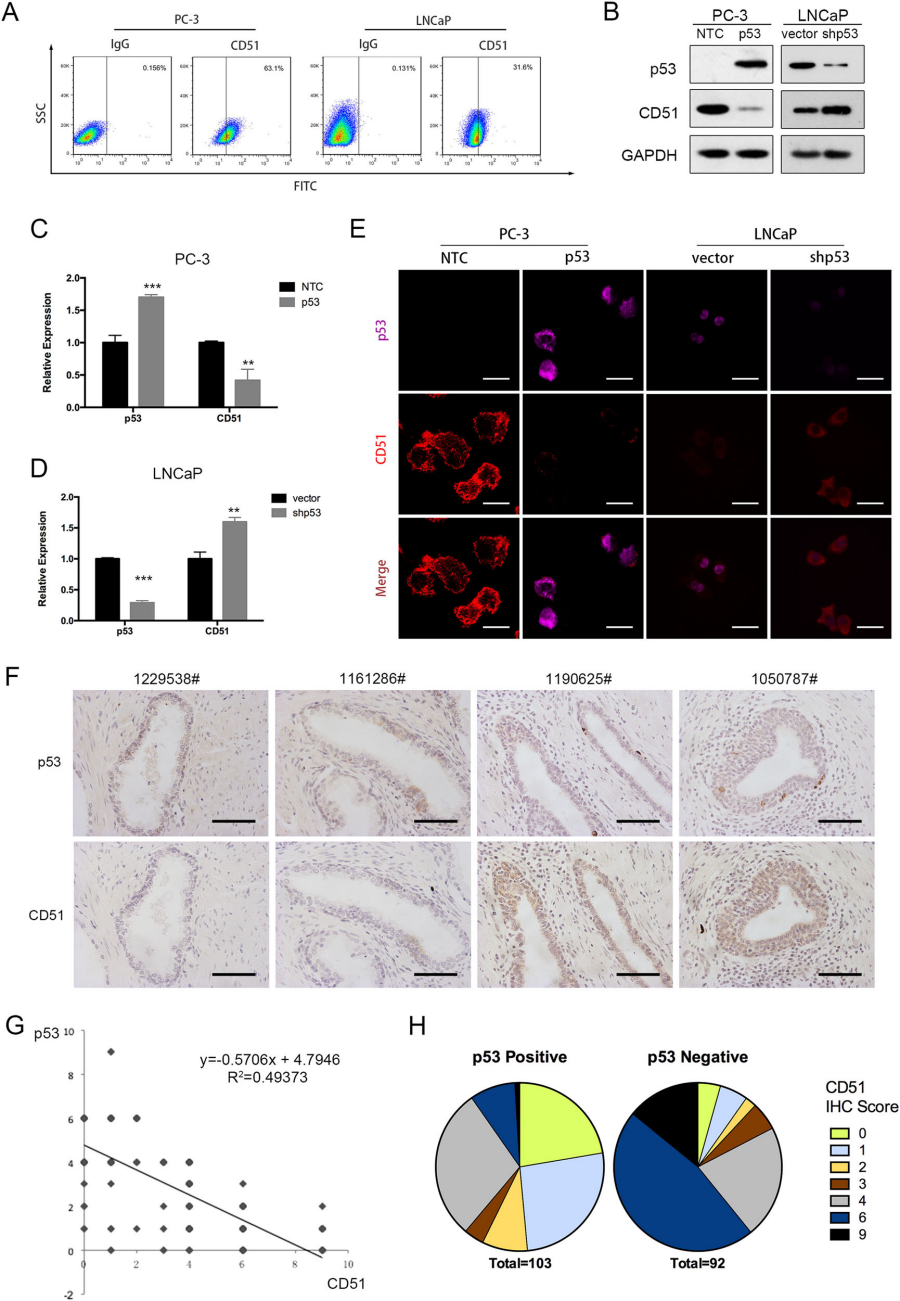
Expression of CD51 was negatively associated with p53. **a** Expression proportion of CD51 in two cell lines measured by FACS, the proportion of CD51+ cells in PC-3 and LNCaP cells was 63.1% and 31.6%, respectively. **b** Immunoblots after overexpression of WT p53 in PC-3 or after knocking down p53 in LNCaP cells. **c**, **d** RT-qPCR was performed to detect the expression of CD51 after the overexpression of WT p53 in PC-3 or after knocking down p53 in LNCaP cells. **e** PC-3 and LNCaP cells were labeled with anti-CD51 (red) and anti-p53 (pink) and analyzed by confocal microscopy. Scale bar: 20 µm. **f** Immunohistochemistry (IHC) results showing that CD51 expression level is negatively associated with p53 expression. Scale bar: 100 μm. **g** Dot plot image showing the association of CD51 and p53 expression in PCa, as determined by Spearman correlation. **h** Pie charts of distinct CD51 IHC scores in the p53-negative and p53-positive human prostate cancer specimens

p53 was reported to repress stemness in many cancers^32, 33^. By knocking down p53 in LNCaP cells, we test the p53-mediated changes in the expression of integrins. Results showed CD51 and the matching β subset (β3 and β6) changed extremely (Fig. S5A). This finding indicates that p53 might limit stemness by downregulating CD51. The PC-3 (p53-null) and LNCaP (p53 wild type, WT) cell lines were used to assess the relationship of different p53 statuses with CD51 expression. We found that PC-3 cells with p53 genomic loss at the p53 locus showed significantly higher CD51 levels than LNCaP cells with WT p53 (Fig. 5a). Approximately 63.1% of PC-3 cells were CD51+, whereas 31.6% were CD51+ in the LNCaP cell lines, respectively. Similarly, Godar et al. reported that p53 inhibits expression of the CD44 cell-surface molecule via binding to a noncanonical p53-binding sequence in the CD44 promoter^34^. We wondered that if there is correlation of p53 with CD51 expression in PCa cell lines. To validate the hypothesis, we measured the expression of CD51 after the knockdown or overexpression of p53. The efficiency of knocking down p53 was detected by immunoblot and RT-qPCR as shown in Fig. S2B and S2C. In PC-3 cells, the overexpression of p53 reduced CD51 expression substantially (Fig. 5b, c). Similarly, the knockdown of p53 in LNCaP cells significantly enhanced CD51 expression (Fig. 5b, d). Next, we confirmed CD51 expression modification after overexpressing or knocking down p53 in two cell lines using confocal microscopy analysis, which is shown in Fig. 5e. To determine if there was a correlation between p53 and CD51 expression within PCa specimens, IHC was performed in matching samples. The results showed that CD51 levels were elevated in p53-negative samples (Fig. 5f). Using Spearman analysis, we found a negative correlation between CD51 and p53 from the same sets of PCa specimens (*r* = 0.7) (Fig. 5g). Moreover, statistical analysis on the CD51 IHC scores of paired p53 WT or p53-loss specimens showed a correlation between high CD51 expression and p53 loss in clinical samples (Fig. 5h). These studies support a direct relationship between the loss of p53 and CD51 expression in PCa.

### p53 represses CD51 transcription in an Sp1/3-dependent manner

**Fig. 6:**
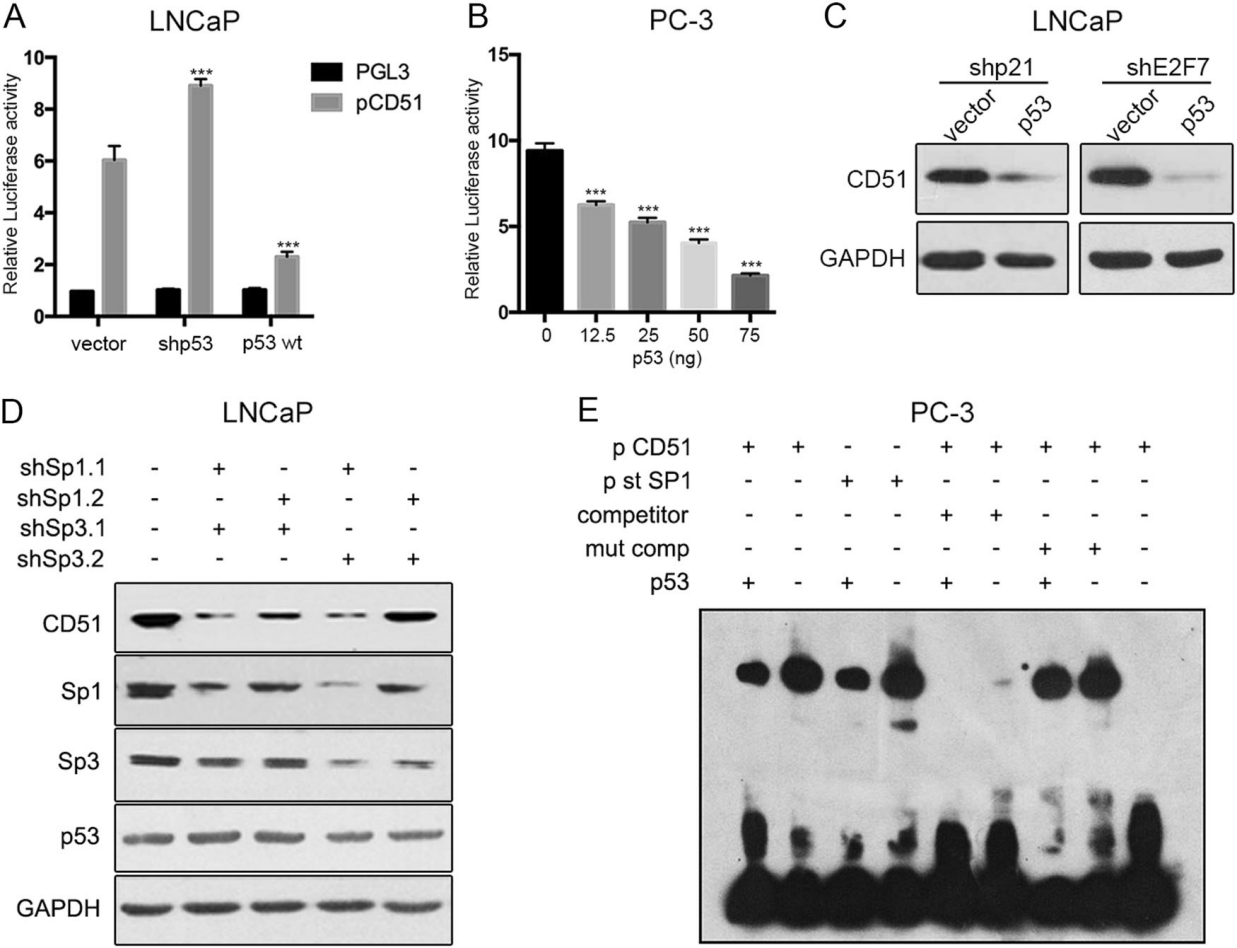
p53 downregulated CD51 expression by antagonizing Sp1 and Sp3. **a** Luciferase assay for analyzing CD51 promoter activity in LNCaP cells with p53 overexpression or knockdown. LNCaP cells with differentially expressed p53 were transfected with the CD51-promoter–reporter construct and PGL3.0-basic vector for 48 h. The luciferase activity is represented as fold-change. **b** PC-3 cells were co-transfected with the CD51-promoter–reporter construct, treated with increasing concentrations of p53 and analyzed for luciferase activity after 24 h. **c** The relationship between CD51 and p53 was detected by immunoblotting. After knocking down p21 and E2F7 and subsequently transfecting the p53 vector into LNCaP cells, the expression of CD51 was detected by immunoblotting. **d** Immunoblot for CD51 after knocking down Sp1 and Sp3 in LNCaP cells. **e** EMSA assays showed that the binding of nuclear proteins to the CD51 promoter was detected in both p53-positive and p53-negative cells. In the competition studies, binding complexes could not be detected; the complex of the nuclear protein with the standard oligonucleotide of the Sp1 binding zone was detected at the same level on the gel. The average was calculated based on three independent experiments. Error bars show the mean ± SD from three technical replicates (two-tailed Student’s *t*-test, ***p *<* 0.001)

To investigate whether p53 influences the CD51 promoter, we transfected LNCaP cells with CD51-promoter–reporter vectors together with either WT-p53-expression vectors or shp53 vectors and found that p53 repressed CD51 promoter activation (Fig. 6a). Similarly, luciferase assays in PC-3 cells showed that increasing the expression of p53 repressed CD51 transcriptional activity (Fig. 6b). Therefore, we demonstrated that p53 decreases CD51 transcription. Previous studies have suggested that p53 is able to repress or activate gene expression by various mechanisms in either a direct or indirect manner. In detail, p53 regulates target gene expression by directly binding to the elements of transcripts^35^, regulating downstream molecules^36^, and antagonizing transcriptional activators^16^. Nevertheless, according to the CHIP-Seq data reported in a recent study, there is no binding site for p53 within the CD51 promoter region^37^. Ho et al. suggested that p53 works indirectly by transcriptionally activating genes such a p21 and E2F7, which ultimately reduces mRNA levels through transcriptional or posttranscriptional mechanisms^36^. Thus, we used immunoblotting to determine whether p53 impacts CD51 in this manner. Indeed, knockdown of p21 or E2F7 had little effect on the expression of CD51 (Fig. S5B). And the repressive effect of p53 is not mediated through p21 or E2F7, because the cells co-expressing potent shRNAs targeting p21 or E2F7 still repressed CD51 in response to p53 (Fig. 6c and S5C). Interestingly, a recent study demonstrated that p53 acts via Sp1/3 to repress Nestin expression^16^. In addition, Czyz et al. identified a sequence in the CD51 promoter that could directly bind Sp1/3^38^. Thus, we hypothesized that p53 represses CD51 through the antagonism of Sp1/3. First, we detected CD51 expression after knocking down Sp1/3 in LNCaP and PC-3 cells. Results showed that co-suppression of both the Sp1/3 genes substantially reduced CD51 expression (Fig. 6d and S5D). Furthermore, we validated that the upregulation of Sp1 and Sp3 rescued cells from the repression of CD51 by p53 (Fig. S5E and S5F). To test if p53 represses CD51 expression by antagonizing Sp1/3 binding to the promoter of CD51, EMSAs were carried out using the 20-bp oligonucleotide corresponding to the region of the CD51 promoter where Sp1/3 might bind. We then characterized the binding of nuclear proteins to the wild-type CD51 promoter, mutant CD51 promoter, and Sp1 standard-binding oligonucleotide using labeled probes with comparable specific activities that were incubated with equivalent amounts of nuclear protein from PC-3 cells (p53 null cell line) with or without transfection of the WT p53 vector. Compared with nuclear proteins from p53-null PC-3 cells, the amount of p53 WT nuclear proteins bound to the CD51 promoter was reduced (Fig. 6e). Our results demonstrated that p53 can reverse CD51 expression by antagonizing Sp1/3 binding to the CD51 promoter.

## Discussion

Metastatic CRPC is a common malignant disease in males and is characterized by poor survival^39^. The molecular mechanisms of hormone therapeutic resistance remain unclear. CSCs in prostate cancer are proposed to be a subpopulation of tumor cells that are important in tumor progression to castration resistance^40^. Identifying new targets for the prevention or treatment of prostate CSCs is therefore of great importance.

Previous studies have provided some CSC markers required for retaining the stem-like status of tumor cells. For example, CD44 (a cell surface marker for CSCs) was shown to enhance the glycolytic phenotype of cancer cells and promote chemoresistance in p53-deficient cells or cells exposed to hypoxia^19^. Raha et al. found that ALDH protected the drug-tolerant subpopulation from the potentially toxic effects of high reactive oxygen species levels and reduced apoptosis^41^. Our results indicate that the population of CD51+ cells is a subset of CD44+ cells and that ALDH expression is much higher in CD51+ cells. These results indicate that CD51 may be a surface marker for prostate CSCs. Our study also shows that CD51 is not only indispensable in maintaining the stem-like characteristics of prostate CSCs, but also serves as a marker for distinguishing CSCs from tumor cells. However, our results do not exclude functions of the β subunit of integrin, which could co-operate with CD51 for tumor progression and metastasis.

p53 has been regarded as a guardian of the genome. p53 has the ability to inhibit chemoresistance^42^, stemness^43^, and metastasis^44^ in cancer. Recent studies have shown that p53 plays an important role in inhibiting CSCs in colorectal cancer^45^, hepatocarcinoma^46^, and acute myeloid leukemia (AML)^32, 47^. Integrins could serve as markers of carcinomas with poor outcomes. Integrins participate in the regulation of cancer stem-cell biology and are required for cancer progression and drug resistance^48^. A recent study on colorectal cancer suggested that tumors with perineural invasion overexpress the CD51 gene when compared to cancers without perineural invasion^49^. These experiments provide evidence that integrins play an important role in stemness. Furthermore, our results demonstrate that there is a positive relationship among the expression of CD51, a high Gleason score, and a low PSA level in PCa patients. We abolished CD51 expression from prostate cancer cells and found that such depletion markedly inhibited tumor initiation and metastasis in vivo and in vitro. Collectively, we proved that CD51 is a stem-associated molecule and that p53 suppresses the stemness, in part, through the inhibition of CD51 expression.

Despite great efforts to elucidate the mechanism by which p53 suppresses stemness and metastasis in cancer, this pathway remains unclear. Some researchers have indicated that p53 inhibits stemness by regulating target microRNA. For instance, p53 increases miR-34 to inhibit stemness in colorectal cancer^44^. However, none of the predicted miR34-binding sites were found in the 3′UTR of the CD51 transcripts (http://regrna.mbc.nctu.edu.tw/index1.php^50^). Others have suggested that p53 restricts stemness in cancer cells by directly targeting downstream molecules. Hong et al. demonstrated that the p53-p21 pathway serves as a barrier in tumorigenicity and iPS cell generation^51^ in a model of p53-null MEF. Carvajal et al. showed that E2F7 is a target gene of p53^52^. Our results show that CD51 is decreased by p53 even if p21 and E2F7 are knocked down. It was previously shown that p53 blocked tumor initiation by inhibiting Nestin, a stem-cell-associated protein, in an Sp1/3 transcription-factor-dependent manner^16^. There is evidence of an Sp1/3 binding site on the CD51 promoter^38^. And a GC-box in the CD51 promoter that is known to bind Sp/Klf transcription factors, especially Sp1 and Sp3^53, 54^. Using luciferase and EMSA assays, we demonstrate that p53 blocks CD51 expression at the pre-transcription stage via Sp1/3. Our findings indicate that p53 can suppress PCa stemness and metastasis by inhibiting the transcription of CD51 in an indirect manner and that the inhibition of CD51 by p53 may be required to effectively restrict PCa progression.

A recent study using limiting-dilution assays and serial tumor transplantation assays showed that PCa cells are endowed with a long-term tumor-propagating capacity. This finding supports the idea that PCa cells harbor self-renewing CSCs and likely represent an important source of CRPC cells^2^. Our data show that the PFS is significantly different between patients in the CD51+ and CD51− group. This finding suggested that CD51 might harbor a subpopulation of stem-like cells in PCa and facilitated patients to recurrence and metastasis.

## Electronic supplementary material

Supplemental Information
