# Supplementary material for: p53-dependent CD51 expression contributes to characteristics of cancer stem cells in prostate cancer
Source: Cell Death Dis. 2018 May 9;9(5):523. doi: 10.1038/s41419-018-0541-x (PMC5943274; doi:10.1038/s41419-018-0541-x)
Supplement: Supplementary file 1 — Supplemental Information [file 41419_2018_541_MOESM1_ESM.docx]

**andud results. mentswere clonedSupplemental Information Figures and Figure Legends**

**Figure S1. Stemness of PCa cells**


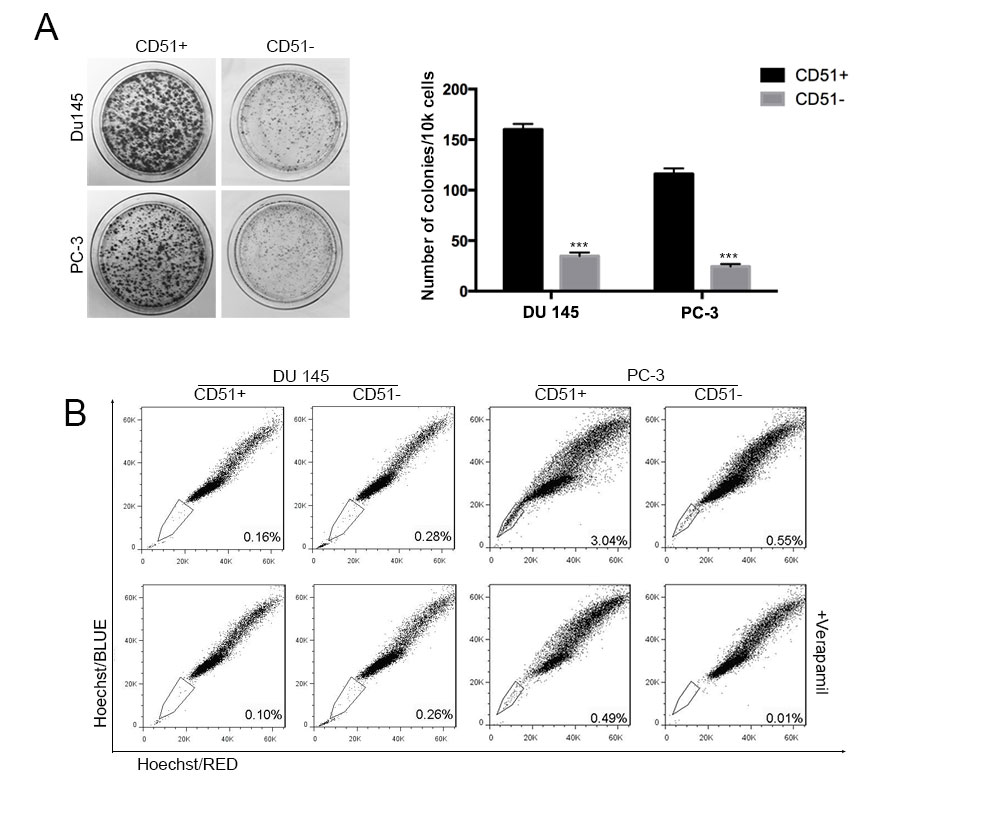


(A) Colony-forming unit (CFU) assay was used to examine the capacity for tumor initiation in CD51+ and CD51- PCa cells. Image of the whole 100 mm dish with 10 × 10^3^ cells initially. The CD51+ subpopulation represented a higher level ability, as evidenced by the number of by CFUs. The numbers of colonies were counted by three different researchers and analyzed using a two-tailed t-test.

(B) The ability to actively exclude the Hoechst 33342 dye, which defines cells as SP cells, was tested by flow cytometry. Compared with the CD51- group, the percentage of SP cells was higher in CD51+ cells in PC-3 group, while SP cells were not found in the DU 145 cell line according to our data. The proportion of SP cells is shown in the image.

**Figure S2 IC50 and the efficiency of knockdown**


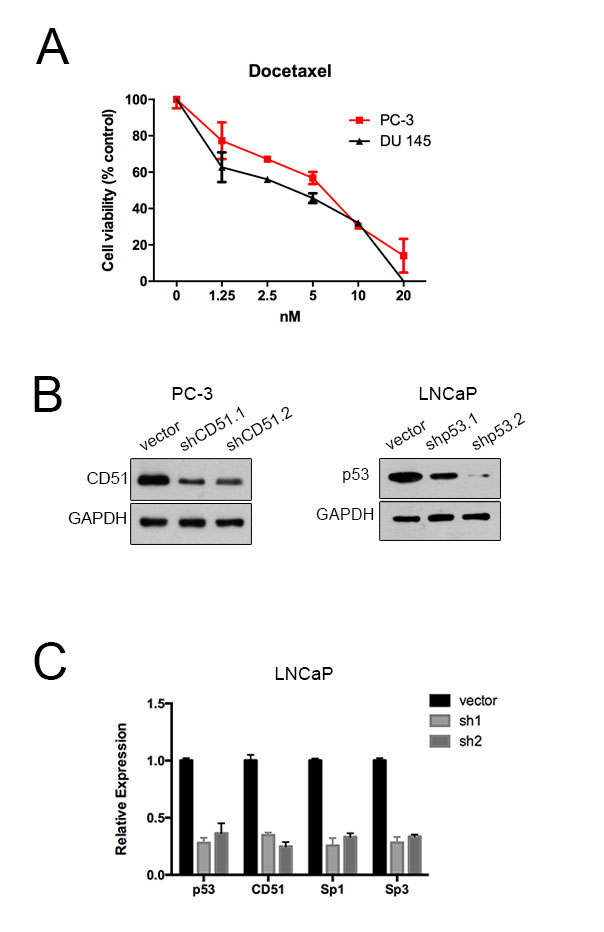


(A) DU 145 and PC-3 cells were treated with docetaxel at various concentrations for 48 hours, and cell viability was measured using the CCK-8 assay. Error bars represent the SD of data obtained from three independent experiments.

(B) The efficiency of knocking down CD51 and p53 was detected in PC-3 (Left) and LNCaP (Right) cells by immunoblotting using specific antibodies. Vectors of shCD51.1 and shp53.2 were clearly effective.

(C) RT-qPCR was used to confirm modification at the mRNA level after knocking down the target gene in LNCaP cells, and the results were analyzed using a two-tailed t-test. Error bars represent the SD from three independent experiments (*p < 0.05, **p < 0.01, ***p < 0.001).

**Figure S3. Knockdown of CD51 in cells reduces the ability of tumor initiation and metastasis in vivo.**


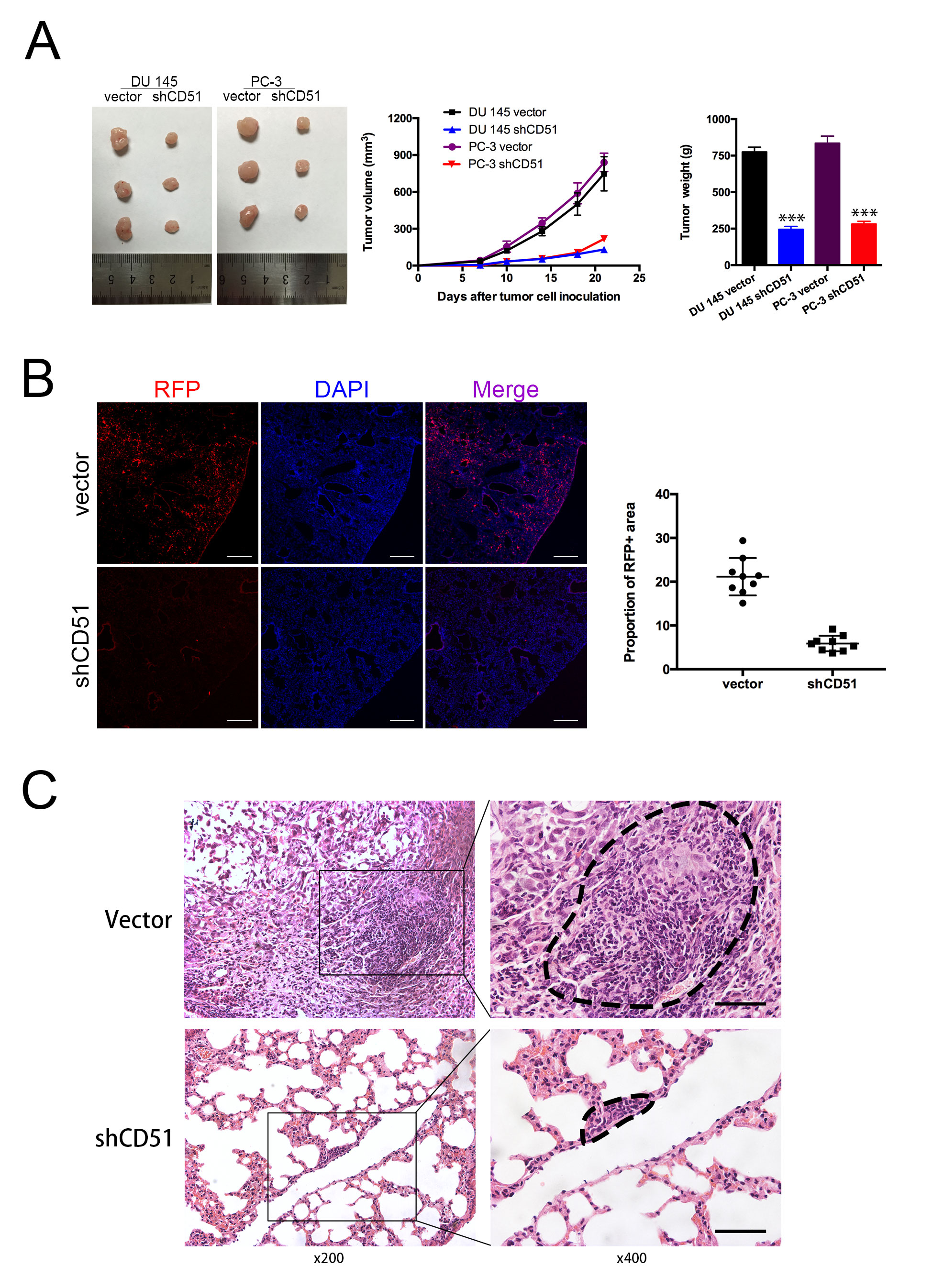


(A) Tumor formation in vivo was induced by subcutaneously injecting 8-week-old SCID mice with PCa cells. Tumors in the right panel were formed by PCa cells with CD51 knocked down, while the tumors in the left panel were formed by cells transfected by the control vector. The tumor weight and volume were subjected to statistical analysis.

(B) Equal numbers of cells with either the RFP-labeled PC-3 control vectors or PC-3 shCD51 vectors were injected into the tail vein of SCID mice. We labeled PC-3 cells with red fluorescent protein (RFP). Mice were killed 6 weeks after cell injection, and the metastatic tumor nodules that had formed in the lungs were examined. Shown are the images of the left lower lobe of the lungs harboring PC-3-RFP cells. Quantification of the RFP positive areas of the whole lung (n = 6) is shown on the right. Scale bar: 200 µm. Testing was repeated in triplicate with different mice.

(C) H&E staining of mice lung after tail vein injection of PC-3 cells. The lung metastasis area was defined with a black dashed line. bar = 100 µm

**Figure S4. Knockdown of CD51 in 22Rv1 cells results in a loss of stem-related properties.**


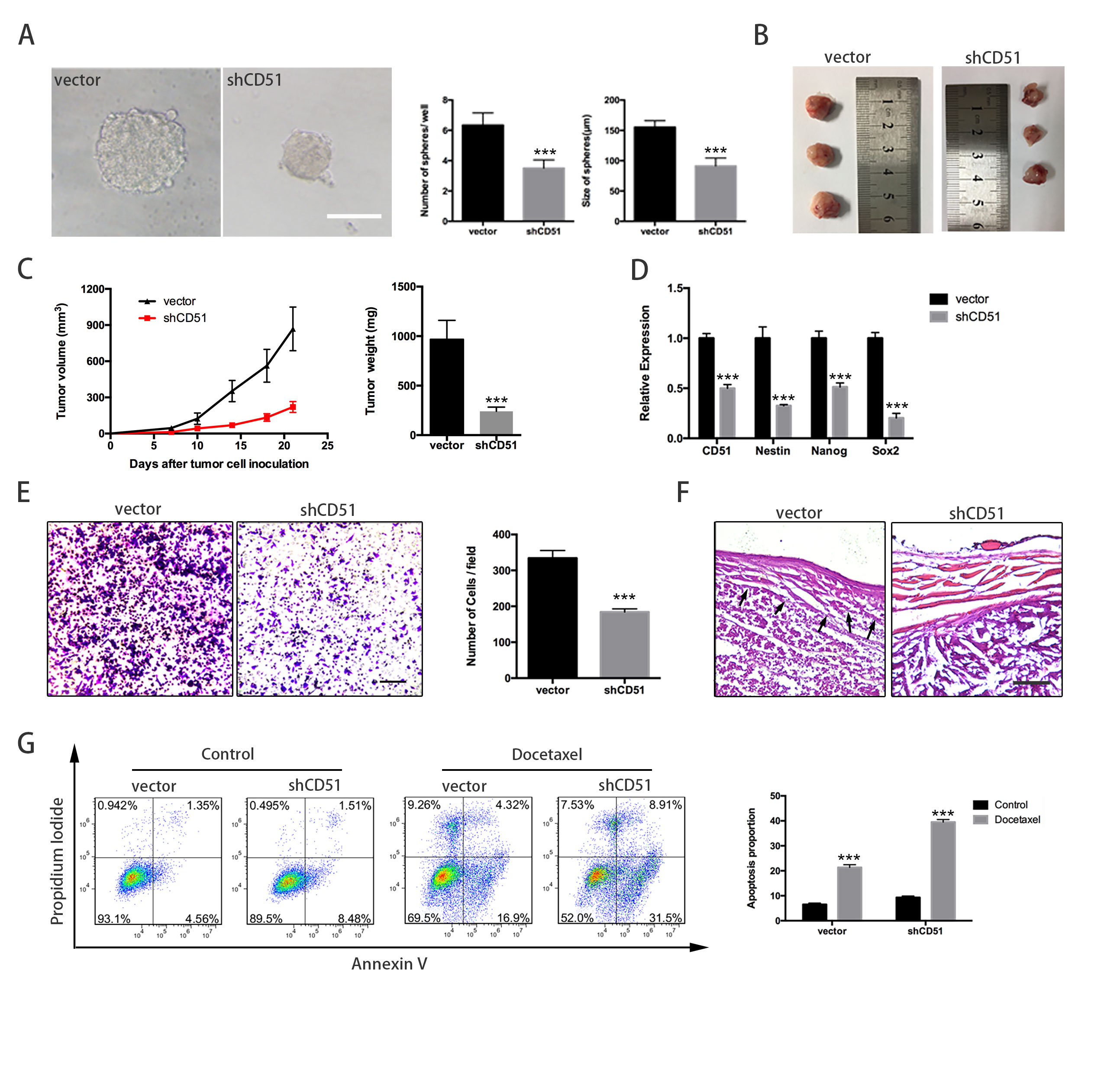


(A) Sphere-formation assays showed that shCD51 decreased the sphere-forming capacities of both cell lines. Scale bar: 100 μm.

(B) Tumor formation in vivo induced by subcutaneously injecting PCa cells into 8-week-old SCID mice. Tumors in the right panel were formed from PCa cells with CD51 knocked down, while the tumors in the left panel were formed from cells transfected with the control vector (n = 8).

(C) The tumor weight and volume were subjected to statistical analysis using two tail t-tests.

(D) RT-qPCR was used to evaluate the mRNA expression levels of the stem-related genes Nestin, Nanog and Sox2 in control 22Rv1 cells and those with CD51 knocked down.

(E) Transwell migration assays showed that the cells transfected with CD51 shRNA had decreased motility compared to the vector controls (left) after 36 h. Scale bar: 200 µm.

(F) H&E staining of tumors formed in SCID mice that were injected subcutaneously with 22Rv1 cells with CD51 knocked down showed a less malignant phenotype in vivo (right). Scale bar: 100 µm.

(G) Drug-resistant assays were performed using flow cytometry to detect the apoptotic proportion (based on annexin V-FITC binding) of cells after treating the cells with docetaxel for 48 hours. Statistical analysis of drug-resistance was performed with a two-tailed t-test.

The average was calculated based on three independent experiments. Error bars show the mean ± SD from three technical replicates (two-tailed Student’s t-test, *p < 0.05, **p < 0.01, ***p < 0.001).

**Figure S5. Correlation between integrins expression with p53, Correlation of CD51 expression with p21, E2F7, Sp1 and Sp3 .**


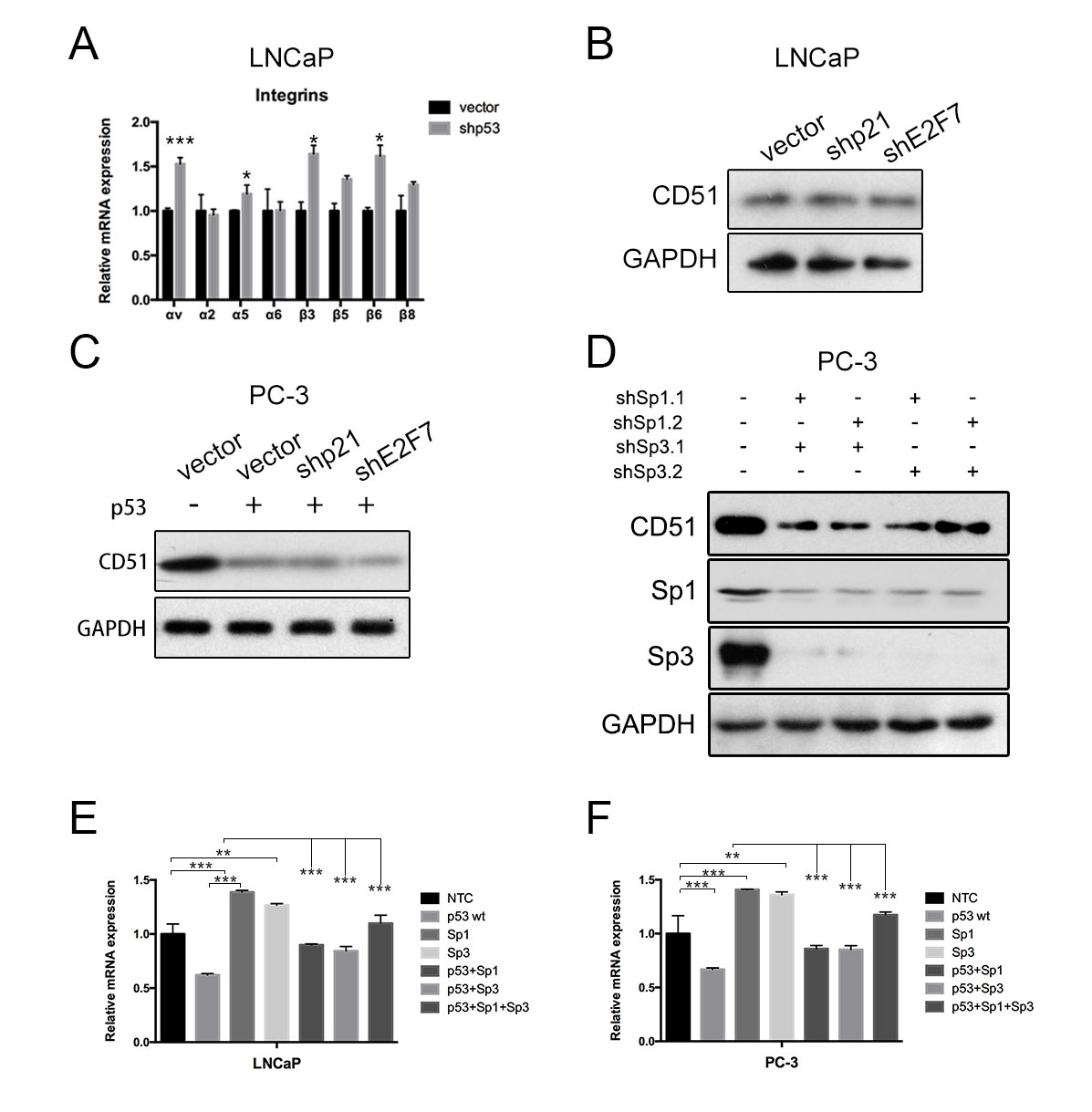


(A) RT-qPCR was used to evaluate the mRNA expression levels of cancer-related integrins in LNCap cells after knocking down p53, and RT-qPCR results were analyzed using a two-tailed t-test. Error bars represent the SD from three independent experiments (**p* < 0.05, **p < 0.01, ***p < 0.001).

(B) Immunoblots for CD51 after knocking down p21 or E2F7 in LNCaP cells.

(C) Immunoblots for CD51 after co-transfected of shp21 or shE2F7 vectors in PC-3 cells with overexpression of p53.

(D) Immunoblot for CD51 after knocking down Sp1 and Sp3 in PC-3 cells.

(E, F) CD51 mRNA expression was examined using RT-qPCR in LNCap (E) and PC-3 cells (F) after transfection with p53, Sp1 and Sp3, either individually or in the designated combination.

Table S1. Primer sequences for the knockdown of specific genes, related to Figures 2 and 6.

|  | Sequences (5'-3') |
| --- | --- |
| shCD51.1 | TGTTGAAGTGTACCCTAGCATTCTTCCTGTCAAAATGCTAGGGTACACTTCAATTTTTTC |
| shCD51.2 | TGTGCAATCTTGTACGTAAAGTCACTTCCTGTCATGACTTTACGTACAAGATTGCACTTTTTTC |
| shp53.1 | TGCACCATCCACTACAACTACATCTTCCTGTCAATGTAGTTGTAGTGGATGGTGTTTTTTC |
| shp53.2 | TGAGGATTTCATCTCTTGTATATGACTTCCTGTCATCATATACAAGAGATGAAATCCTTTTTTTC |
| shSp1.1 | TGACCTGGAGTGATGCCTAATATCTTCCTGTCAATATTAGGCATCACTCCAGGTTTTTTTC |
| shSp1.2 | TGTTCTATATTATTATATATATATACTTCCTGTCATATATATATATAATAATATAGAATTTTTTC |
| shSp3.1 | TGCGCGAGATGATACTTTGATTACTTCCTGTCATAATCAAAGTATCATCTCGCGTTTTTTC |
| shSp3.2 | TGTTCTATTTTTATATTTTAAAATGCTTCCTGTCACATTTTAAAATATAAAAATAGAATTTTTTC |
| shp21.1 | TTCACTGTCTTGTACCCTTGTCTTCCTGTCAACAAGGGTACAAGACAGTGATTTTTTC |
| shp21.2 | TTCACTGTCTTGTACCCTTGTCTTCCTGTCAACAAGGGTACAAGACAGTGATTTTTTC |
| shE2F7.1 | TTCACTGTCTTGTACCCTTGTCTTCCTGTCAACAAGGGTACAAGACAGTGATTTTTTC |
| shE2F7.2 | TTGCCCAAGAAACCCTCAGATTCTTCCTGTCAAATCTGAGGGTTTCTTGGGCATTTTTTC |

Table S2. Primer sets used in this study, related to Figures 2, 4 and S1.

| Name |  | Sequences (5'-3') |
| --- | --- | --- |
| GAPDH | Forward | GGAGCGAGATCCCTCCAAAAT |
|  | Reverse | GGCTGTTGTCATACTTCTCATGG |
| TP53 | Forward | GAGGTTGGCTCTGACTGTACC |
|  | Reverse | TCCGTCCCAGTAGATTACCAC |
| CD51 | Forward | ATCTGTGAGGTCGAAACAGGA |
|  | Reverse | TGGAGCATACTCAACAGTCTTTG |
| Nanog | Forward | TTTGTGGGCCTGAAGAAAACT |
|  | Reverse | AGGGCTGTCCTGAATAAGCAG |
| Sox2 | Forward | GCCGAGTGGAAACTTTTGTCG |
|  | Reverse | GGCAGCGTGTACTTATCCTTCT |
| Nestin | Forward | CTGCTACCCTTGAGACACCTG |
|  | Reverse | GGGCTCTGATCTCTGCATCTAC |
| p21 | Forward | CGATGGAACTTCGACTTTGTCA |
|  | Reverse | GCACAAGGGTACAAGACAGTG |
| E2F7 | Forward | AAAGGGACTATTCCGACCCAT |
|  | Reverse | ACTTGGATAGCGAGCTAGAAACT |
| Integrin β3 | Forward | AGTAACCTGCGGATTGGCTTC |
|  | Reverse | GTCACCTGGTCAGTTAGCGT |
| Integrin β5 | Forward | GGAAGTTCGGAAACAGAGGGT |
|  | Reverse | CTTTCGCCAGCCAATCTTCTC |
| Integrin β6 | Forward | CTCAACACAATAAAGGAGCTGGG |
|  | Reverse | AAAGGGGATACAGGTTTTTCCAC |
| Integrin β8 | Forward | GTGAAAGTCATATCGGATGGCG |
|  | Reverse | GCTATCAAGAGCGAGATGAGACG |
| Integrin α6 | Forward | CAGTGGAGCCGTGGTTTTG |
|  | Reverse | CCACCGCCACATCATAGCC |
| Integrin α2 | Forward | GGGAATCAGTATTACACAACGGG |
|  | Reverse | CCACAACATCTATGAGGGAAGGG |
| Integrin α5 | Forward | GGCTTCAACTTAGACGCGGAG |
|  | Reverse | TGGCTGGTATTAGCCTTGGGT |
